# Genomic loci associated with Fusarium stalk rot resistance and related agronomic traits in maize

**DOI:** 10.1007/s00122-026-05290-x

**Published:** 2026-06-22

**Authors:** Desmond Darko Asiedu, Bettina Kessel, Benedict Oyiga, Patrick Thorwarth, Thomas Presterl, Thomas Miedaner

**Affiliations:** 1 State Plant Breeding Institute, Universität Hohenheim, Stuttgart, Germany; 2 Kleinwanzlebener Saatzucht (KWS) KWS SAAT SE & Co. KGaA, Einbeck, Germany; 3 Cluster of Excellence GreenRobust, University of Hohenheim, Stuttgart, Germany

## Abstract

**Key message:**

Two novel QTLs conferring Fusarium stalk rot resistance were identified in the Kemater flint landrace population, thereby broadening the genetic base for FSR resistance breeding. Given the polygenic inheritance of Fusarium stalk rot resistance, genomic selection could be explored as a potential strategy for improving resistance to stalk rot in maize.

**Abstract:**

Fusarium stalk rot (FSR) is one of the most prevalent and destructive soil-borne diseases of maize, caused by *Fusarium graminearum* (teleomorph *Gibberella zeae*), which leads to substantial yield losses worldwide. This study investigated the genetic basis of FSR resistance in the Austrian “Kemater Landmais Gelb” (KE) population under field conditions. A total of 180 KE testcrosses were generated by crossing 180 randomly chosen KE doubled-haploid lines with a dent tester line. These testcrosses were evaluated following artificial inoculation with *F. graminearum* using needle injection, and disease severity was assessed based on internode proportion, a robust phenotyping scale for FSR. Field trials were conducted using an alpha-lattice design with two replications across two locations over two years. Moderate-to-high broad-sense heritability estimates (*H*^2^ = 0.48–0.82) were realized for FSR severity, plant height, and days to silking. Low–moderate phenotypic (*r* =  − 0.17 to -0.22) and genotypic correlations (*r*_g_ = − 0.22 to − 0.31) were found between FSR severity and agronomic traits. Strong linkage disequilibrium (LD) was observed between the QTL, ZmSYNBREED_65065_837, associated with days to silking and all QTLs for plant height (*r* $\sim 0.85$). This high LD could explain the observed correlations between these traits. Genome-wide association analysis in the KE testcross population identified two QTLs that jointly explained 21% of the genotypic variance in FSR severity. Several putative candidate genes located within these QTL regions were associated with pathogen recognition, signal transduction, and defense responses to FSR infection. Once the stability and functionality of these QTLs and candidate genes are established, these loci will provide valuable insights into the molecular mechanisms underlying FSR resistance and serve as useful targets for genomic selection in maize breeding programs.

**Supplementary Information:**

The online version contains supplementary material available at 10.1007/s00122-026-05290-x.

## Introduction

Given the global scale of maize production, yield losses can lead to significant economic impacts. Fusarium stalk rot (FSR) of maize predominantly caused by *Fusarium graminearum* sensu stricto Schwabe (teleomorph *Gibberella zeae*) is one of the most devastating maize diseases around the world, including Europe and North America (Starkey et al. 2007; Pfordt et al. 2020b; Asiedu et al. 2024). *F. graminearum* is aggressive in causing FSR in Central Europe by thriving in higher precipitation and moderate temperature conditions and this aligned with methodological studies of FSR, comparing different *Fusarium* pathogens and inoculation methods (Pfordt et al. 2020a; Asiedu et al. 2024). A survival mechanism of *F. graminearum* is that it exploits weeds and other crops as alternative hosts serving as primary sources of inoculum. Aside *F. graminearum*, which belongs to *Fusarium graminearum* species complex, other members such as *F. bothii, F. meridionale, F. cortaderiae,* and *F. austroamericanum* cause stalk rot in maize in South Africa and South America (Castañares et al. 2016; Beukes et al. 2018; Del Ponte et al. 2022). Reports show that *F. equiseti*, *F. proliferatum*, *F. verticillioides*, *F. temperatum,* and *F. subglutinans* also may cause maize stalk rot in Germany, Switzerland (Dorn et al. 2011; Pfordt et al. 2020a, b), France, Poland, Belgium (Czembor et al. 2015; Boutigny et al. 2017; Coninck et al. 2019), and China (Gai et al. 2017), however, to a lesser extent. The typical symptoms of FSR in a susceptible maize begin with a reduction in the enzymatic activities of peroxidase, polyphenol oxidase, and phenylalanine ammonia lyase (Xue et al. 2020, 2021; Yang et al. 2023). This continues with a reduction in soluble sugars in the plant system, an increase in senescence, a decline in stalk strength, and increase in stalk lodging, which eventually leads to yield loss. On a global scale, FSR causes 4.5% yield loss on average (Savary et al. 2019). Stem rot exacerbates corn lodging in the context of increasing thunderstorms and heavy rainfall events resulting from climate change (Barten et al. 2022).

Conventional management practices had seen limited success in combating FSR in maize (Asiedu and Miedaner 2025). Hence, breeding resistant varieties is the most sustainable option of tackling FSR disease in maize breeding pipelines when the disease is persistent. However, phenotyping FSR disease in maize is tedious. Due to the complexity and polygenic inheritance of FSR resistance in maize, we developed a more robust and reproducible system for phenotyping FSR resistance in maize (Asiedu et al. 2024). We recommended needle injection of *F. graminearum* conidia and internode proportion, i.e., summing up the percentages of visible infection (mycelium and/or necrosis) of affected internodes, in a longitudinally split maize stalk, as an ideal quantitative scale to discriminate maize genotypes on different levels of FSR resistance (Asiedu And Miedaner 2025).

Classical quantitative genetics have shown that FSR resistance is primarily controlled by many minor-effect loci with additive effects (Yang et al. 2010; Ma et al. 2017; Rashid et al. 2022; Song et al. 2024a, b; Asiedu And Miedaner 2025). A recent literature review identified a total of 46 minor QTLs mapped across the whole maize genome except chromosome 9 (Asiedu And Miedaner 2025). Since then, four novel QTLs were identified and validated in a Chinese inbred line on chromosomes 5, 8, and 10, of which the major QTL on chromosome 8 was fine mapped (Zhang et al. 2026). Additionally, four major-effect QTLs, *qRfg1*, *qRfg2*, *qRfg3,* and *Rgsr8.1* on genomic bins 10.03/4, 1.09/10, 3.06/7, and 8.06/8 were mapped for FSR resistance in maize. We noticed that chromosome 1 is a hub for quantitative disease resistances as it contained ~ 33% of the entire minor-effect FSR QTLs (Asiedu And Miedaner 2025) as well as disease resistance genes underlying Gibberella ear rot, common smut, gray leaf spot, southern corn leaf blight, northern corn leaf blight, and Stewart’s wilt (Wisser et al. 2006; Chung et al. 2010; Jamann et al. 2016; Böhm et al. 2017).

Maize landraces could act as reservoirs of valuable resistance alleles and beneficial agronomic traits, offering breeders a means to expand the genetic diversity of elite maize germplasm and secure long-term genetic improvement in breeding programs. European flint maize landraces are a prime example of this potential (Böhm et al. 2014). This include “Kemater Landmais Gelb” (KE, originating from Austria) and “Petkuser Ferdinand Rot” (PE, originating from Germany), which captures a higher proportion of genetic diversity and are adapted to a wide geographical range in Central Europe (Mayer et al. 2017; Hölker et al. 2019). KE landrace population is reported to contain several quantitative disease resistance QTLs including ear rot resistance in maize (Han et al. 2016, 2018; Gaikpa et al. 2021). Additionally, a QTL meta-analysis for maize ear rot conducted by Akohoue and Miedaner (2022) demonstrated how diverse resistance sources, such as the KE landrace, contributed to identifying 40 meta-QTLs containing multiple putative resistance genes. Previous reports indicate that double-haploid (DH) lines and their testcrosses derived from the European flint maize landraces have significant potential for improving elite germplasm, particularly with respect to Gibberella ear rot resistance (Akohoue et al. 2021), and grain and silage traits (Brauner et al. 2019).

Therefore, the exploitation of KE landraces’ genetic diversity could tackle the adverse effects of FSR on maize. Here, we want to dissect the genetic architecture of FSR resistance in the 180 testcrosses sourced from landrace KE DH lines. Specifically, we aimed to (i) estimate the variance components and heritability of FSR severity given as internode proportion affected and the related agronomic traits days to silking, and plant height, (iii) estimate phenotypic and genotypic correlations between FSR severity and agronomic traits across four environments (location × year combinations), and (iii) detect significantly associated SNPs from a 600 k SNP array and putative candidate genes underlying FSR resistance via genome-wide association study (GWAS).

## Materials and methods

### Plant materials, field experiments, and data collection

Plant materials were composed of 180 testcrosses produced from a cross between the “Kemater Landmais gelb” (KE) flint DH population and an elite dent line (tester) provided by KWS SAAT SE & Co. KGaA, Einbeck, Germany. The DH lines represent a random sample of the DH lines from KE as described by Hölker et al. (2019). We phenotyped 180 testcrosses plus 20 standard checks in Hohenheim (HOH) near Stuttgart, Germany, and Einbeck (EIN) in 2024 and 2025. The experimental design was 20 × 10 alpha-lattice design (10 genotypes per 20 incomplete blocks) with two replicates in both locations and years. Within the experimental design, 5 rows of KWS dent line were grown adjacent to the testcrosses and the 20 standard checks were fully randomized within the testcrosses. Each plot was a single row of 3 m length with an inter-row spacing of 0.75 m and intra-row spacing of 15 cm and 20 kernels. Sowing was done mechanically.

Ten plants per row, excluding border plants on each side were inoculated with inoculum prepared from the highly aggressive *F. graminearum* (Fg) isolate FG 163 at a concentration of 4 × 10^6^ spores/mL. Each maize stalk was inoculated by a one-needle vaccinator on the second internode from soil level with 2 ml of the inoculum at silk emergence. To prevent the liquid from running out, wounds created by the needle injection were closed using a vaseline cream that is harmless to the fungus as well as the plant. Days to silking (DS), plant height (PH, cm), and FSR severity (%) assessed using internode proportion were recorded in 4 environments (= location × year combinations). Days to silking were recorded as the number of days taken to achieve ≥ 50% female flowering per plot. PH was measured plot-wise from ground level to the first tassel branch using a meter rule in cm. At physiological maturity, FSR severity was collected by estimating for each internode, the proportion covered by mycelium and/or necroses (0–100%) and summing up those individual proportions across all infected internodes for each plant. For each internode, 0% refers to no visible sign of mycelium and 100% indicates that the internode is fully covered by mycelium and/or necroses. The full phenotyping protocol has been published previously (Asiedu et al. 2024). Within this experiment, a maximum of two lowest internodes was affected. Two independent raters participated in the scoring process, with each evaluator assessing one replicate of the alpha-lattice design, which consisted of two replicates per location. This approach was implemented to minimize raters’ bias and to ensure an unbiased estimation of experimental error in the mixed-model analysis.

### Phenotypic data analysis

#### Single-row analysis

The single plant data from ten plants per row for each genotype for each trait were inspected to remove outliers, which met at least two of these four robust outlier detection methods: Tukey’s interquartile range test, robust z-score, Grubbs’ test, and Rosner’s test (Grubbs 1949; Rosner 1975; Tukey 1977; Rousseeuw And Hubert 2011). In this field studies, we realized the outliers were just data-entry errors, broken plants, and insect damage unrelated to FSR disease infection. The excluded outliers within each row for each environment were $\sim$ 5% of each environment raw data.

#### Plot-level analysis

For the separate and combined environment analysis in models 1 and 2, the plot means for outlier-free single plant data were estimated and explored to remove outliers using Bonferroni–Holm test to judge residuals standardized by the re-scaled mean absolute deviation (Method M4; Bernal-Vasquez et al. 2016) for the analysis of each trait. Following the mixed-model analysis output, the residuals for both individual and combined environments were roughly normally distributed after careful inspection of the quantile–quantile plot of the residuals. A mixed linear model was first fitted for all three traits within each of the four environments (= location × year combinations) using ASReml-R package version 4.1 (Butler et al. 2017) as follows:

1 $$y_{ijk} = \mu + g_{i} + \, r_{j} + \, b_{k(j)} + \, e_{ijk}$$

where *y*_*ijk*_ = phenotype of the *i-*th genotype within the *k-*th block nested within the *j-*th replicate, *µ* = common intercept*, g*_*i*_ = effect of *i-*th genotype, *r*_*j*_ = effect of *j-*th replicate, *b*_*k(j)*_ = effect of the *k-*th incomplete block nested within the *j-*th replicate, and e_*ijk*_ = the residual error for observation *y*_*ijk*_, assumed to be independent and normally distributed with mean 0 and a constant variance ${\sigma}_{\mathrm{e}}^{2}$; i.e., ${e}_{ijk} \sim N(0, {\sigma}_{\mathrm{e}}^{2})$. Based on model 1, the genotypic values of the testcrosses were computed as best linear unbiased estimations (BLUEs) within each of the four environments. Genotype and replicate effects were fitted as fixed effects to estimate the BLUEs within all locations.

2 $$y_{ijkl} = \mu + g_{i} + \, e_{j} + \, ge_{ij} + \, r_{k(j)} + b_{l(jk)} + \varepsilon_{ijkl}$$

where *y*_*ijkl*_ = phenotypic response of the trait for combined environments, *g*_*i*_ = effect of *i-th* genotype, *e*_*j*_ = effect of *j-th* environment, *ge*_*ij*_ = the interaction effect between *i-*th genotype and *j-*th environment, *r*_*k(j)*_ = the effect of the *k-th* replication within *j-*th environment, *b*_*l(jk)*_ = effect of the *l-th* incomplete block within each *k-th* replication and *j-*th environment, and *ε*_*ijkl*_ = residual error term in model associated with separate error variances for individual environments. Based on model 2, the genotypes were fitted as a fixed effect, whereas the rest of the terms were fitted as random to estimate the BLUEs across the four environments. The BLUEs of the KE testcrosses from model 2 were then used in the GWAS analysis.

3 $$y_{ijkl} = \mu + e_{j} + x_{i} [g_{i}^{c} + (ge)_{ij}^{c} \left] { + \left( {1 - x_{i} } \right)} \right[g_{i}^{t} + \left( {ge)_{ij}^{t} } \right] + \, r_{k(j)} + b_{l(jk)} + \varepsilon_{ijkl}$$

where *y*_*ijkl*_ = phenotypic response of the trait, *µ* $=$ common intercept, *g*_*i*_ = effect of *i-th* genotype, *e*_*j*_ = effect of *j-th* environment, ${x}_{i}$ $=$ dummy variable taking the value 1 if the *i-th* genotype is a standard check and 0 if it is KE testcross*,* the effect of the *i-th* genotype is denoted *by* ${g}_{i}^{c}$ (if it is a standard check) *or* ${g}_{i}^{t}$ (if it is a KE testcross), the interaction effect between the *i-th* genotype and the *j-th* environment *is* denoted *by* ${ge}_{ij}^{c}$ (standard check) *or* ${ge}_{ij}^{t}$ (KE testcross)*, r*_*k(j)*_ = the effect of the *k-th* replication within *j-th* environment, *b*_*l(jk)*_ = effect of the *l-th* incomplete block within each *k-th* replication within *j-th* environment, and *ε*_*ijkl*_ = residual error term in the model connected with different error variances for individual environments. A dummy indicator ${x}_{i}$(check vs KE testcross) was used to fit separate random-effect variance components for checks and KE testcrosses (Piepho et al. 2006) based on model 3. All the terms in model 3 except the intercept were fitted as random to extract the variance components of all terms using the ASReml-R package version 4.1 (Butler et al. 2017). The likelihood ratio test (LRT) was implemented to analyze significance of variance components (Morrell 1998). Broad-sense heritability (*H*^*2*^) according to Cullis et al. (2006) was estimated as follows:

$$H^{2} = \left( {1 - \frac{{\overline{v}^{{{\mathrm{BLUP}}}} }}{{2{\upsigma}_{{\mathrm{g}}}^{{2}} }}} \right)$$

where ${\sigma}_{g}^{2}$ is the genotypic variance and ${\overline{v} }^{\mathrm{B}\mathrm{L}\mathrm{U}\mathrm{P}}$ is the average standard error of difference of two genotypic best linear unbiased predictions (BLUPs). The BLUPs and genotypic variance (${\sigma}_{g}^{2}$) of the KE testcrosses were extracted from model 3 and used in Cullis’ heritability estimates for all respective traits.

### Phenotypic and genotypic correlations of traits

Pearson’s product moment correlation coefficients (*r*) between BLUEs of traits from combined environments were computed as phenotypic correlations using *cor.test* in R programming language in R version 4.3.1 (R Core Team 2023). Bivariate models as detailed in Wilson et al. (2010) for each pair of traits were fitted with an unstructured variance–covariance matrix using *corgh* option for genetic effects of testcrosses, while environment main effects, genotype–environment interaction, replicates, and blocks were modeled with *diag* option to model trait-specific variances. Residuals were fitted with environment-specific variances to accommodate heterogeneity in different environments in ASReml-R package version 4.1.

### Molecular marker analysis

The 180 parental DH lines were genotyped using a high-density Affymetrix® Axiom® Maize Genotyping Array optimized for temperate maize (Mayer et al. 2020). Genotypic data were processed using a combination of PLINK version 1.9 (Purcell et al. 2007; Chang et al. 2015) and BEAGLE version 5.1 (Browning et al. 2018). Quality control filtering was conducted in PLINK to remove variants with a genotype call rate below 80%, a minor allele frequency (MAF) below 5% and heterozygosity > 5%. Following quality control, genotype phasing and imputation were performed with BEAGLE to improve genotype accuracy by leveraging haplotype information within defined genomic windows. In addition, markers that were in linkage disequilibrium with each other were determined based on SNP pruning parameters (window size = 50, step size = 50, LD threshold, *r*^*2*^ = 0.3). The filtering procedures reduced the number of SNPs from 500,786 to 19,751 polymorphic SNPs.

### Genome-wide association analysis

The BLUEs of the 180 testcrosses (indexed according to the names of their parental lines) together with 19,751 polymorphic SNP markers were used for GWAS of FSR severity using the BLINK model implemented in the *gapit* function of the R package Genome Association and Integrated Prediction Tool (GAPIT) 3.4.0 (Wang and Zhang 2021). To account for potential population structure, principal components (PCs) were included as fixed covariates in the GAPIT implementation of the BLINK model. Ten PCs were initially evaluated, and the optimum number of PCs was selected based on Bayesian information criterion (BIC) values (Schwarz 1978), resulting in seven PCs being retained in the final GWAS analysis (Table S1). In addition, a genomic relationship matrix (GRM) was estimated from the 19,751 SNP markers using the VanRaden method (VanRaden 2008). The BLINK model does not directly incorporate a genomic relationship matrix or kinship matrix as a random effect. Therefore, in this study, the GRM was used only to assess and visualize genetic relatedness among the KE parental DH lines through a kinship heatmap (Fig. S2). BLINK only controls false-positive associations by iteratively selecting pseudo-QTNs and applying linkage disequilibrium-based marker filtering within a fixed-effect regression approach. The BLINK method performed the analysis in two-step fixed-effect model approach (FEM I and FEM II), combined with a filtering process, which occurred iteratively (Huang et al. 2019).

In BLINK, the filtering process involves testing and ranking all polymorphic SNPs based on their p values. The *t* most significant SNPs referred to as pseudo-QTNs are selected according to a Bonferroni-corrected threshold defined in Eq. (4), ensuring that they are not in high linkage disequilibrium (LD) with each other (Pearson correlation, *r* < 0.7). In FEM I, the term ${S}_{ij}{d}_{j}$ is used for testing the remaining markers individually. In FEM II, all *t* pseudo-QTNs selected after filtering are jointly re-evaluated using the Bayesian information criteria (BIC) as an optimization criterion. The BIC was calculated as twice the negative log-likelihood plus the penalty on the number of free parameters included in the model as follows:

$$\mathrm{B}\mathrm{I}\mathrm{C}$$ = -$$2LL$$ + $$2kLn(n)$$

where *LL* is log-likelihood; *k* is number of pseudo-QTNs; *Ln* is the natural logarithm; and n is the number of individuals. Subsequently, the top *k* out of *t* pseudo-QTNs with the lowest BIC values are selected and included as covariates in FEM I to test the remaining markers.

The two fixed-effect model equations are as follows:

FEM I: $${y}_{i}$$ = $${S}_{i1}^{*}{b}_{1}$$  + $${S}_{i2}^{*}{b}_{2}$$+……+ $${S}_{ik}^{*}{b}_{k}$$ + $${S}_{ij}{d}_{j}$$  + $${e}_{i}$$

FEM II: $${y}_{i}$$ = $${S}_{i1}^{*}{b}_{1}$$ + $${S}_{i2}^{*}{b}_{2}$$  + *…*+ $${S}_{ik}^{*}{b}_{k}$$  + $${e}_{i}$$

where ${y}_{i}$ = BLUEs across the 4 environments on the *i-th* genotype,${ S}_{i1}^{*}$, ${S}_{i2}^{*}$,…${S}_{ik}^{*}$ are genotype scores of *k* pseudo-QTNs, which is initiated as an empty set, and ${b}_{1}, {b}_{2},$….$,{b}_{k}$ are the corresponding effects of pseudo-QTNs. ${S}_{ij}$ denotes the *i-th* genotype and the *j-th* marker; ${d}_{j}$ is the respective effect of the *j-th* marker; and ${e}_{i}$ is the residual with ${e}_{i}\sim$ *N* (*0,*${\sigma}_{e}^{2}$*)*.

To identify which SNPs were most likely associated with each trait in the Manhattan plot, we adopted Bonferroni-corrected threshold of –log10 (*p *value) = 4.86. The respective *p *value was calculated as follows:

4 $$p - {\text{value }} = \frac{\alpha }{{n_{{{\mathrm{informative}}}} }}$$

where $\alpha$ = 0.05 is type 1 error rate and ${n}_{\mathrm{i}\mathrm{n}\mathrm{f}\mathrm{o}\mathrm{r}\mathrm{m}\mathrm{a}\mathrm{t}\mathrm{i}\mathrm{v}\mathrm{e}}$ is the total number of informative or effective SNPs extracted from the *simpleM* algorithm (Gao et al. 2008; Johnson et al. 2010). The *simpleM* method quantifies the effective number of independent SNPs (*M*ₑff) by applying principal component analysis to the SNP correlation matrix. Because many SNPs were correlated through linkage disequilibrium, they carry redundant genetic information. The algorithm identifies the smallest set of independent principal components that together explain 99.5% of the total genetic variance. In this study, 3,657 effective SNPs captured 99.5% of the genotypic variation in the testcross population and were used to adjust the Bonferroni correction, providing a less conservative significance threshold. The total proportion of genotypic variance (*p*_*G*_) explained by the significant SNPs was estimated using the formulas (Utz et al. 2000):

$${P}_{G}$$ = $$\frac{{\mathrm{R}}^{2}\mathrm{a}\mathrm{d}\mathrm{j}.}{{H}^{2}}$$

where the ${\mathrm{R}}^{2}\mathrm{a}\mathrm{d}\mathrm{j}.$ is the adjusted ${\mathrm{R}}^{2}$ and ${H}^{2}$ is the broad-sense heritability of the trait. The adjusted ${\mathrm{R}}^{2}$ was computed from a multiple linear regression model of the significant SNPs extracted from the GWAS analysis. The multiple linear regression model is as follows:

5 $$y={s}_{i}+{s}_{j}+{s}_{k}+...+{s}_{p}+e$$

where *y* is the BLUEs of 4 environments for respective trait and ${s}_{i}$ + ${s}_{j}$  + ${s}_{k}$+…+${s}_{p}$ denote the SNP markers included in the model. The individual SNPs were sequentially added to the fitted model based on the magnitude of their p values in descending order of strength of association, ${s}_{i}$ > ${s}_{j}$> ${s}_{k}$  > …… ${s}_{p}$ (Gaikpa et al. 2020; Akohoue et al. 2022). The term $e$ is the residual error of the fitted linear model. Independent *p*_*G*_ values were derived from the sum of squares of each SNP marker (${SS}_{\mathrm{s}\mathrm{n}\mathrm{p}}$) from the analysis of variance table of the linear model and computed as follows:

$${p}_{G}$$ = $$\left(\frac{{SS}_{\mathrm{s}\mathrm{n}\mathrm{p}}}{{SS}_{\mathrm{T}\mathrm{o}\mathrm{t}\mathrm{a}\mathrm{l} }{\times H}^{2}}\right)$$  × 100.

where ${SS}_{\mathrm{s}\mathrm{n}\mathrm{p}}$ is the sum of squares of each individual SNP, ${SS}_{\mathrm{T}\mathrm{o}\mathrm{t}\mathrm{a}\mathrm{l}}$ is the total sum of squares of the linear model and the additive effect of each significant SNP by fitting one SNP at a time as explained by Würschum et al. (2015).

### LD decay statistics

LD decay was estimated using the quality-controlled, unpruned SNP dataset to investigate LD structure. SNPs used for LD decay met call rate > 80%, MAF > 5% and heterozygosity < 5% and not subjected to LD pruning, as pruning generates an approximately linkage-equilibrated marker set and may bias LD estimates. Pairwise LD (*r*^2^) was calculated for SNP pairs within 1 Mb, and mean *r*^2^ values were summarized in 1-kb distance bins. These computations were done in PLINK accompanied by the libraries *ggplot2* and *data.table* in R software version 4.3.1.

### Candidate gene detection

We examined genomic regions within 250 kb upstream and downstream of the two significant FSR linked SNPs (namely, ZmSYNBREED_10522_344 on chromosome 1 and ZmSYNBREED_51479_551 on chromosome 6) to identify potential candidate genes, using the publicly available B73 reference genome version 4 (Zm-B73-REFERENCE-GRAMENE-4.0, Jiao et al. 2017) on the maize genome database (Woodhouse et al. 2021). The SNPs were annotated using MaizeGDB (https://www.maizegdb.org/) and NCBI (https://www.ncbi.nlm.nih.gov/) resources. Although genome-wide LD approached background levels at approximately 561 kb, a more conservative ± 250-kb interval was selected to balance LD extent with mapping resolution and to reduce the inclusion of unrelated genes. This approach is consistent with previous studies demonstrating that LD extent varies among maize breeding populations and directly affects the effective resolution of association mapping (Van Inghelandt et al. 2011; Han et al. 2016; Rashid et al. 2022; Song et al. 2024a; Zhou et al. 2024).

## Results

### Effect of environmental conditions on FSR severity

**Fig. 1:**
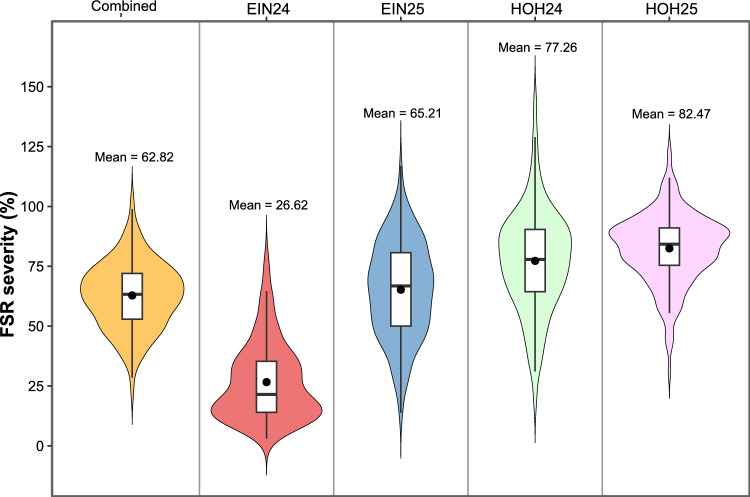
Violin plots showing the BLUEs for Fusarium stalk rot (FSR) infection in Kemater testcrosses at Hohenheim (HOH) and Einbeck (EIN) in 2024 and 2025 and with the four environments combined. HOH24, EIN24, HOH25, EIN25 = individual location × year environment with HOH = Hohenheim, EIN = Einbeck followed by the experimental year. Filled black dots represent the mean of FSR severity, and a horizontal dash depicts the median

Weather conditions at HOH were warmer than at EIN in both years (Table 1). Temperatures at both locations were higher in 2024 than in 2025. While HOH received more rain than EIN in 2024, this pattern reversed in 2025, with EIN recording greater precipitation. Differences in FSR severity between HOH and EIN were larger in 2024 but minimal in 2025 (Fig. 1). In both years, FSR severity was higher at HOH, indicating that HOH provides a more conducive environment for FSR infection than EIN, and this was evident in our observations at both locations.

**Table 1:** Temperature and precipitation readings for Hohenheim (HOH) and Einbeck (EIN), Germany, for the growth period (May–October) in 2024 and 2025

Element	HOH		EIN	
	2024	2025	2024	2025
*Temperature (*$^\circ{\rm C}$*)*				
Mean	16.43	16.18	15.81	15.36
Max	20.70	20.20	19.19	18.31
Min	11.45	10.50	10.35	11.20
*Precipitation (mm)*				
Mean	99.55	69.60	83.64	139.54

### Phenotypic and genotypic variation of FSR resistance and agronomic traits

FSR disease infections were noticed in all four environments. HOH25 experienced the highest FSR severity compared to all other environments with the lowest in EIN24 (Fig. 1). However, this environment had the lowest genetic variation (Table 2) in KE testcrosses compared to the other three environments. Einbeck had lower FSR severities than Hohenheim in both years. Moderate-to-high repeatabilities ranging from 0.48 to 0.63 were noticed in each environment. FSR severity had a broad-sense heritability of 0.72 across all environments, and high H^2^ was observed for both PH and DS. We found significant genotype, environment, and genotype–environment interaction variances across all environments (*p* ≤ *0.001*) except PH, which showed no significant genotype × environment interaction variance. On average, KE testcrosses were 35 cm taller in Einbeck than in Hohenheim across both years. In Einbeck, DS was similar in both years but for Hohenheim, KE testcrosses produced silks 3 days earlier in 2025 than in 2024. Low albeit significant phenotypic and genotypic correlations were found between FSR severity and DS or PH (Table 3). Therefore, no correction for any of these traits was necessary. Correlations between DS and PH were moderate.

**Table 2:** Summary statistics, variance components (${\sigma }^{2}),$ and repeatability (*R*) estimates for one-environment analyses and parameters including broad-sense heritability (*H*^2^) estimates for combined analyses for Fusarium stalk rot (FSR) severity (%), plant height (PH, cm), and days to silking (DS, days) of 180 testcrosses within and across four environments

Traits	Parameter	Environments				
		HOH24	EIN24	HOH25	EIN25	Combined
FSR Severity (%)	Max	144.45	81.17	123.11	116.95	103.36
	Min	19.82	2.98	30.91	13.79	22.19
	${\sigma}_{G}^{2}$	277.78^**^	170.00^**^	112.73^**^	270.89^**^	152.29^**^
	${\sigma}_{GE}^{2}$	–	–	–	–	30.29^**^
	${\sigma}_{\upvarepsilon }^{2}$	315.16	152.95	180.80	205.67	235.25
	*R/H*^2^	0.54	0.61	0.48	0.63	0.72
PH (cm)	Max	317.53	361.20	259.55	294.86	302.28
	Min	241.41	271.35	186.80	220.15	237.31
	Mean	285.91	324.05	223.59	254.82	272.03
	${\sigma}_{G}^{2}$	131.08^**^	167.99^**^	144.60^**^	127.82^**^	124.51^**^
	${\sigma}_{GE}^{2}$	–	–	–	–	8.97
	${\sigma}_{\upvarepsilon }^{2}$	106.73	129.75	85	88.16	116.82
	*R/H*^2^	0.61	0.63	0.68	0.64	0.79
DS (days)	Max	85.09	82.46	81.94	82.46	84.03
	Min	73.30	72.48	70.24	72.48	73.55
	Mean	78.70	77.04	75.51	77.04	77.81
	${\sigma}_{G}^{2}$	2.84^**^	2.41^**^	4.06^**^	5.72^**^	2.89^**^
	${\sigma}_{GE}^{2}$	–	–	–	–	0.39^**^
	${\sigma}_{\upvarepsilon }^{2}$	1.88	0.54	1.05	2.01	1.62
	*R/H*^2^	0.65	0.80	0.78	0.74	0.82

HOH, EIN = Hohenheim, Einbeck, respectively; the numbers represent the year

${\sigma}_{G}^{2}$ = genotypic variance, ${\sigma}_{GE}^{2}$ = genotype-by-environment interaction variance,

${\sigma}_{\varepsilon }^{2}$= residual variance, *H*^2^ = broad-sense heritability. ** Significant at *p* ≤ *0.001*. The absence of asterisk (*) indicates that variance components are not statistically significant at this *p* value

**Table 3:** Phenotypic (above the diagonal) and genotypic (below the diagonal) correlations between Fusarium stalk rot (FSR) severity (%), plant height (PH), and days to silking (DS) for 180 testcrosses across four environments

Trait	FSR severity	PH	DS
FSR severity		− 0.17^*^	− 0.22^*^
PH	− 0.22*		0.42^*^
DS	− 0.31*	0.51*	

^*^Significant at *p* ≤ 0.05

### Principal component analysis

To determine the appropriate number of principal components (PCs) for correcting population structure, we initially evaluated ten PCs and selected the best number of PCs with the highest Bayesian information criterion (BIC). This investigation indicated that seven PCs, collectively explaining 25.94% of the molecular variation in the KE testcross population (Table S1), were optimal for inclusion in the GWAS model. PCA plot with the first two PCs together explaining a low molecular variance of 12.04% further revealed three small subclusters (Fig. S1a) within the KE population. We incorporated seven PCs to effectively account for the population structure (Fig. S1b, c) to reduce the likelihood of false-positive associations.

### LD decay response in KE population

**Fig. 2:**
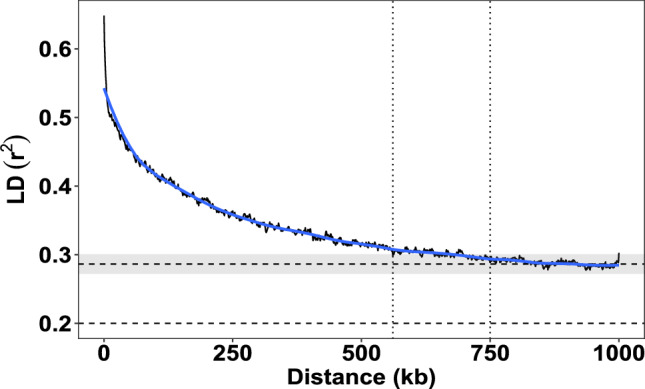
LD decay plot in KE parental DH population for FSR severity

Genome-wide LD declined sharply with increasing physical distance between SNPs (Fig. 2). Mean *r*^2^ values were greater than 0.5 at short distances and decreased progressively before reaching a plateau at larger genomic intervals. The background LD level, estimated as the median mean *r*^2^ across the final 200 kb of the distance range, was approximately *r*^*2*^ $=$ 0.286. Mean LD approached this background level at approximately 561 kb, beyond which additional decline was minimal. Mean *r*^2^ did not decline to 0.20 within the 1-Mb window evaluated, and the convergence of LD to background levels near ~ 561 kb at *r*^2^ $=$ 0.3 provided an empirical estimate of the genomic distance beyond which LD is no longer strongly distance-dependent in this KE parental DH population. This pattern is expected for structured breeding germplasm, where background LD can persist over extended genomic intervals.

### QTL discovery for FSR severity

**Fig. 3:**
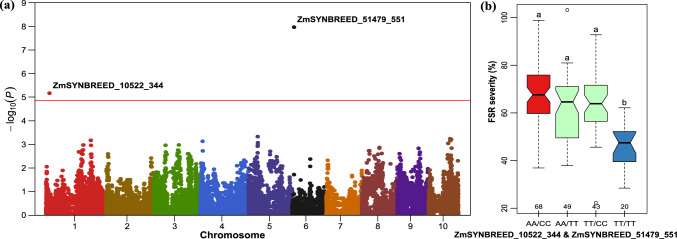
**a** Manhattan plot for GWAS discovery for FSR severity (%) in 180 testcrosses of “Kemater Landmais gelb” population across four environments. **Bonferroni-corrected threshold** at *p* ≤ *0.05* (shown as red horizontal line) **b** Box plots of stacked two significant SNPs associated with FSR severity (AA, CC = susceptible alleles for ZmSYNBREED_10522_344 and ZmSYNBREED_51479_551, respectively, TT, resistance allele for both SNPs)

We identified two QTLs in the KE testcross population across four environments that jointly explained approximately 21% of the genotypic variation in FSR severity (Fig. 3a, Table 4). One QTL was located on chromosome 1 (ZmSYNBREED_10522_344) and the other on chromosome 6 (ZmSYNBREED_51479_551), with both surpassing the Bonferroni-corrected significance threshold. Two significant QTLs were detected for PH on chromosome 8 (ZmSYNBREED_65326_781 and ZmSYNBREED_65128_528), both meeting our exploratory threshold. For DS, two QTLs were found on chromosome 8 (ZmSYNBREED_66242_938 and ZmSYNBREED_65065_837) exceeding the Bonferroni-corrected threshold with the remaining two SNPs on chromosome 3 (ZmSYNBREED_31239_115) and chromosome 8 (ZmSYNBREED_65628_540) meeting our exploratory threshold. Moreover, the QTLs for DS, ZmSYNBREED_65065_837 (*r* $\sim 0.85$), had strong LD with all the QTLs associated with PH. This could explain moderate correlations between PH and DS. Both significant SNPs for FSR severity are acting additive together, when combined (Fig. 3b). Among the resulting testcrosses, 20 KE lines had both resistance alleles showing a significantly (*p* $<$ *0.05*) reduced FSR severity (46.3%) compared to the 68 lines with two susceptible alleles (67.3%). Interestingly, both resistance alleles together had a higher disease reduction than either of the resistance alleles alone. The FSR resistance QTL on chromosome 1 contained four plausible candidate genes, including a leucine-rich protein and an ethylene-responsive element binding protein, both previously associated with FSR-related defense responses (Table S2). In contrast, the QTL on chromosome 6 comprised three candidate genes whose roles in FSR resistance are unvalidated, except for an iron-binding protein reported to respond to biotic stress signals.

**Table 4:** Significant SNPs for Fusarium stalk rot (FSR) severity (%), plant height (PH, cm), days to silking (DS, days), chromosomal position (Pos.), *P* value, minor allelic frequency (MAF), additive effect (Add.), and proportion of explained genotypic variance (*P*_*G*_) in “Kemater Landmais gelb” (KE) testcrosses across four environments

Trait	Marker	Chr	Pos	P value	MAF	Add	^*a*^*P*_*G*_	^*b*^*P*_*G*_
FSR severity	ZmSYNBREED_10522_344	1	17,832,835	6.46E−06	0.35	− 4.89	7.59	9.73
	ZmSYNBREED_51479_551	6	8,517,925	1.08E−08	0.38	− 4.97	12.33	12.33
	${P}_{G}$_total_							20.74
PH	ZmSYNBREED_65326_781	8	136,382,986	3.48E−05	0.24	4.75	14.22	14.22^ns^
	ZmSYNBREED_65128_528	8	129,537,446	4.84E−05	0.24	4.70	14.72	0.51^ns^
	${P}_{G}$_total_							13.44
DS	ZmSYNBREED_31239_115	3	17,366,005	2.42E−04	0.06	− 0.83	10.89	10.25
	ZmSYNBREED_66242_938	8	169,171,639	1.10E−07	0.05	− 1.31	7.74	7.74
	ZmSYNBREED_65065_837	8	126,490,600	1.25E−05	0.30	0.51	20.80	18.53
	ZmSYNBREED_65628_540	8	146,285,544	2.58E−05	0.10	0.82	24.00	14.14
	${P}_{G}$_total_							49.02

NB: ^a^Genetic variances obtained from single fit of individual QTLs, ^b^Genetic variances obtained from joint fit of individual QTLs, and ${P}_{G}$_total_ = Total proportion of genotypic variance from joint fit of the two QTLs computed with adjusted *R*^2^. All the genotypic variances of QTLs fitted in the multiple linear regression were significant at *p* < 0.001 apart from QTLs fitted for PH which were nonsignificant (ns)

## Discussion

Previous investigations into the genetic and phenotypic basis of FSR severity in different maize populations revealed that FSR resistance is controlled by a few major-effect QTLs and many minor-effect QTLs with moderate heritability. KE landrace population had already been used to detect separate QTLs associated with Gibberella ear rot resistance (Gaikpa et al. 2021) but not for FSR resistance. In this study, we examined KE testcrosses for FSR resistance via GWAS and explored the covariance between the agronomic traits, plant height, and days to silking with FSR severity. Hölker et al. (2019) identified three clusters corresponding to DH lines derived from three European maize landraces, Kemater Landmais Gelb (Austria), Petkuser Ferdinand Rot (Germany), and Lalin (Spain) via principal coordinate analysis. A closer inspection of the Kemater cluster showed the presence of smaller subgroups within this population. Similarly, Gaikpa et al. (2021) reported smaller clusters within the KE population based on structure analysis. Therefore, we did not exclude the presence of subpopulation within the KE DH parental lines in our GWAS. To account for this possibility, we investigated the population structure of this same KE population and performed GWAS both with and without correction for population structure to evaluate its potential impact on our results. Based on this assessment, population structure within the KE parental DH population was included as a correction factor in our final GWAS analysis.

### Covariance between FSR severity with agronomic traits in KE testcrosses

We noticed high FSR severity across all environments and years except EIN24. Environmental conditions and their interactions exerted a pronounced influence on the aggressiveness of *Fusarium graminearum* ability in causing stalk rot disease. The high genotype–environment interaction relative to the high genotypic variance for FSR severity indicates that environmental effects played a major role in determining the resistance of KE testcrosses to FSR infection. These large variance components were cumulatively reflected in the moderate to high broad-sense heritability (H^2^) estimates for FSR severity. High weather variability in temperate regions contribute to the genotype–environment interaction variances (Pè et al. 1993; Ma et al. 2017; Liu et al. 2021). It is therefore necessary for plant breeders to implement multi-environment phenotyping for reliable estimation of genetic values for FSR resistance in maize.

From a physiological perspective, previous studies have shown that the severity of FSR infection in maize is strongly influenced by plant growth stage and its interaction with biotic stresses (Dodd 1980; Sobek And Munkvold 1999; Smith And White 2015). Additionally, we investigated genotypic and phenotypic correlation patterns between the agronomic traits PH and DS and FSR severity. Aside from the high repeatabilities and broad-sense heritabilities of these agronomic traits (Table 2), we found significant low-to-moderate phenotypic (*r* = − 0.17 to − 0.22) and genotypic correlations (*r*_g_ = − 0.22 to − 0.31) between FSR severity and PH and DS, respectively.

Notably, our results are consistent with the findings of Asiedu et al. (2024) who reported negative correlations between DS and FSR severity (*r* $=-\hspace{0.17em}$0.17), and this indicates that increased susceptibility to FSR is associated with earlier flowering although we inoculated all genotypes at the same date. This could possibly be one of the reasons why Hohenheim showed a higher FSR infection than Einbeck in 2025 (Table 2). Similarly, a GWAS study on GER resistance in a KE DH population reported comparable associations (Gaikpa et al. 2021). We conclude that the consistent pattern of negative correlations among DS, FSR severity, and GER severity is influenced by both the environmental conditions and the genetic background of the maize germplasm evaluated in the field experiment.

### GWAS revealed two genetic regions linked to FSR resistance

Our GWAS supports the hypothesis that FSR is governed by numerous small-effect loci, each contributing only marginally to the overall genotypic variance. The unexplained variance in the KE testcrosses is therefore likely attributable to a plethora of minor-effect loci additively interacting. Previous studies have shown that the statistical power of GWAS is strongly influenced by sample size, allele frequency, and the proportion of phenotypic variance explained by each locus (Korte And Farlow 2013), with relatively small populations often having limited power to detect minor-effect QTL. Moreover, the genotypic variances of QTLs with small-to-moderate effects estimated from small populations can be overestimated due to the Beavis effect (Beavis 1998; Xu 2003). Wang and Xu (2019) also showed that statistical power increases substantially with increasing sample size and QTL effect size in GWAS analysis, whereas small-effect loci require considerably larger populations for reliable detection. As result, we further suspected that the modest population size (*N* = 180) reduced the statistical power of our GWAS to detect a larger number of significant loci. Therefore, increasing population size and applying robust multi-locus GWAS models are recommended to improve the discovery of QTLs conferring FSR resistance.

Interestingly, all the QTLs detected within this field experiment were within the chromosomal positions previously reported to be associated with FSR resistance (Asiedu And Miedaner 2025). In particular, the identification of minor QTL on chromosome 1 was expected, as multiple studies have consistently reported minor-effect loci on chromosome 1 (Yang et al. 2010; Rashid et al. 2022; Song et al. 2024a, b). Although, chromosome 1 ranks second to chromosome 6 in the number of minor QTLs for FSR resistance, it contains numerous quantitative resistance loci for other diseases (Wisser et al. 2006; Chung et al. 2010; Böhm et al. 2014; Jamann et al. 2016; Duan et al. 2019). We therefore recommend that plant breeders critically examine this genomic region and prioritize the evaluation of candidate genes for FSR resistance.

We discovered another QTL on chromosome 6. This chromosome was known to cover a high density of minor-effect loci with reported *PVE* spanning from 2.09 to 8.42% for FSR resistance in previous studies (Yang et al. 2004; Rashid et al. 2022; Song et al. 2024a, b). Notably, the biallelic C/T SNP in ZmSYNBREED_51479_551 on chromosome 6 is the same in the SNP *S6_136411611* located on genomic bin 6.05 on a different physical position, and this was previously reported to be associated with resistance to both *Fusarium verticillioides* and *F. graminearum* in a *non-Tuxpeño* testcross population evaluated under tropical conditions in GWAS (Song et al. 2024b).

Moreover, a recent work by Zhang et al. (2026) identified several genomic regions associated with stalk rot resistance. In contrast, the QTLs detected in this study for FSR resistance did not overlap with the physical intervals reported in their study. This shows that the loci uncovered here represent additional sources of resistance. The differences in these studies are expected, due to the different genetic backgrounds and mapping strategies employed. Zhang et al. (2026) used a biparental RIL population for linkage mapping, whereas our study evaluated testcross performance in a DH‑derived population using GWAS. Additionally, the two studies relied on different phenotyping approaches, and FSR severity scored on a 0–100% scale versus the number of discolored internodes, which can affect the genetic signals detected. Together, these dissimilarities between studies depict the value of integrating diverse germplasm, mapping populations, and phenotyping methodologies to broaden the catalog of loci contributing to stalk rot resistance.

Finally, to avoid unexplainable errors and/or failure of QTL introgression in elite germplasm for FSR resistance in maize breeding programs, we advocate that these two significant QTLs should be validated in different genetic backgrounds and multiple environments. As shown here, they are not colocated with QTLs for the agronomic traits, PH, and DS.

### Candidate genes for FSR resistance

The candidate genes covering the two significant SNPs (Table 4) for FSR resistance included leucine-rich transmembrane protein kinase 1 (LTK1), an electron carrier or iron-binding protein (FeBP), B3 domain-containing protein (ABIVP1 transcription factor), and ethylene-responsive element binding protein 2 (EREBP2) (Table S2). These genes are functionally involved in protein serine or threonine kinase activity, protein binding, ATP binding, protein phosphorylation, response to stress, positive regulation of DNA-templated transcription, and DNA binding. LTK1 resides within the transmembrane domain of the leucine-rich repeat receptor-like kinase (LRR-RLK) family, whose members bind to extracellular molecules and activate intracellular kinase domains through phosphorylation and autophosphorylation. This receptor activation transmits external signals across the plasma membrane to initiate pathogen defense, growth, and nutrient-signaling pathways in response to biotic and abiotic stresses (Schlessinger 2000; Hubbard And Miller 2007; An et al. 2022; Shi et al. 2023; Sun et al. 2023). In maize, LTK1 plays a crucial role in pathogen recognition and immune signaling. Gao et al. (2025) investigated the LRR-RLK gene family during their study of *Fusarium verticillioides* infection in maize at different time periods and identified 205 members distributed across 15 subfamilies, 96% of which contain transmembrane domains. Several LRR-RLKs were upregulated during early infection, showing rapid pathogen detection, with protein activation occurring in the middle and later developmental phases of maize. LRR-RLKs signaling events elevate the expression of defense-related genes, such as pathogenesis-related protein genes and phytohormone biosynthetic regulators, thereby increasing immune responses to *F. verticillioides*. Similarly, the functional response of LRR-RLK subgroups (leucine-rich repeat protein kinase family protein and leucine-rich repeat extension-like protein 3) have been reported to contribute to resistance against *F. graminearum* stalk rot in maize (Liu et al. 2021; Song et al. 2024b). In contrast, for B3 domain-containing proteins, no substantial literature currently supports their involvement in signaling activities for FSR infection. FeBP, an iron-binding protein, shares their characteristic with the candidate gene *GRMZM2G070323* on chromosome 1, identified in the FSR study by Song et al. (2024a). *GRMZM2G070323* is involved in protein phosphorylation, a process that helps crops respond to stresses by coordinating the expression of functional genes (Yao and Xu 2017). EREBP2, a member of the AP2/EREBP transcription factor superfamily, belongs to the APETALA2/ethylene-responsive factor (AP2/ERF) family. These transcription factors act as key regulators of plant hormone signaling by three mechanisms: Their transcript levels are hormone-regulated, they modulate hormone sensitivity and gene expression through synergistic or antagonistic interactions, and they control hormone biosynthesis or metabolism via feedback loops (Nolan et al. 2017; Xie et al. 2019; Viswanath et al. 2023; Tang et al. 2024). In maize, infection by the hemibiotrophic fungal pathogen *Fusarium graminearum* triggers AP2/EREBP-mediated signaling responses. This leads to the upregulation of hormonal pathways and the release of salicylic acid (SA). The deposition of SA subsequently induces the production of maize terpenoid phytoalexins. These phytoalexins strengthen maize plant defense, enabling it to resist *F. graminearum*-mediated stalk rot infection (Qi et al. 2023).

In summary, the two SNPs detected here are found in genomic regions containing plausible candidate genes with functional roles in pathogen recognition, signal transduction, and transcriptional regulation of defensive responses to FSR infection in maize genotypes. Our results support the fact that FSR resistance is a complex, polygenic trait regulated by a series of coordinated defense pathways. It is therefore advisable to validate the function of these candidate genes using transcriptomics to provide molecular evidence supporting their involvement in FSR resistance in both susceptible and resistant maize genotypes. In addition, targeted genome-editing technologies such as CRISPR/Cas9 can be employed to further validate the roles of these candidate genes in FSR resistance through gene knockout, modification, or overexpression approaches.

## Conclusions

This study established the molecular basis of FSR resistance in KE landrace population and examined the potential covariation between FSR severity and related agronomic traits. The existence of significant genotype–environment interaction observed in field evaluations suggests the need for multi-environment testing when assessing maize genotypes for FSR resistance. The genetic diversity within the KE landrace highlights it as an important resource for plant breeders to introgress FSR resistant alleles into their maize breeding pipelines. Hence, establishing a strong correlation between elite flint lines and testcross performance for FSR resistance will be indispensable to identify superior lines. Such maize lines can then be advanced across multiple locations and years to ensure stable resistance to stalk rot. Given that the KE population already contains QTLs for Gibberella ear rot resistance (Gaikpa et al. 2021), together with the two QTLs associated with FSR resistance identified in the present study, these loci could serve as valuable targets for marker-assisted pyramiding into elite flint germplasm after further validation in independent populations and environments. In the future, these loci may also contribute to genomic-assisted breeding strategies aimed at improving resistance to Fusarium stalk rot disease in different genetic backgrounds in maize.

## Supplementary Information

Below is the link to the electronic supplementary material.

Supplementary file1 (PDF 944 KB)

## Data Availability

Data will be made available upon request.
