# Supplementary material for: Genomic loci associated with Fusarium stalk rot resistance and related agronomic traits in maize
Source: Theor Appl Genet. 2026 Jun 22;139(7):186. doi: 10.1007/s00122-026-05290-x (PMC13287220; doi:10.1007/s00122-026-05290-x)
Supplement: Supplementary file 1 — Supplementary file1 (PDF 944 KB) [file 122_2026_5290_MOESM1_ESM.pdf]

Genomic loci associated with Fusarium stalk rot resistance and related agronomic traits in maize

Desmond Darko Asiedu[1], Bettina Kessel[2], Benedict Oyiga[2], Patrick Thorwarth[1,3], Thomas Presterl[2] and Thomas Miedaner[1*]

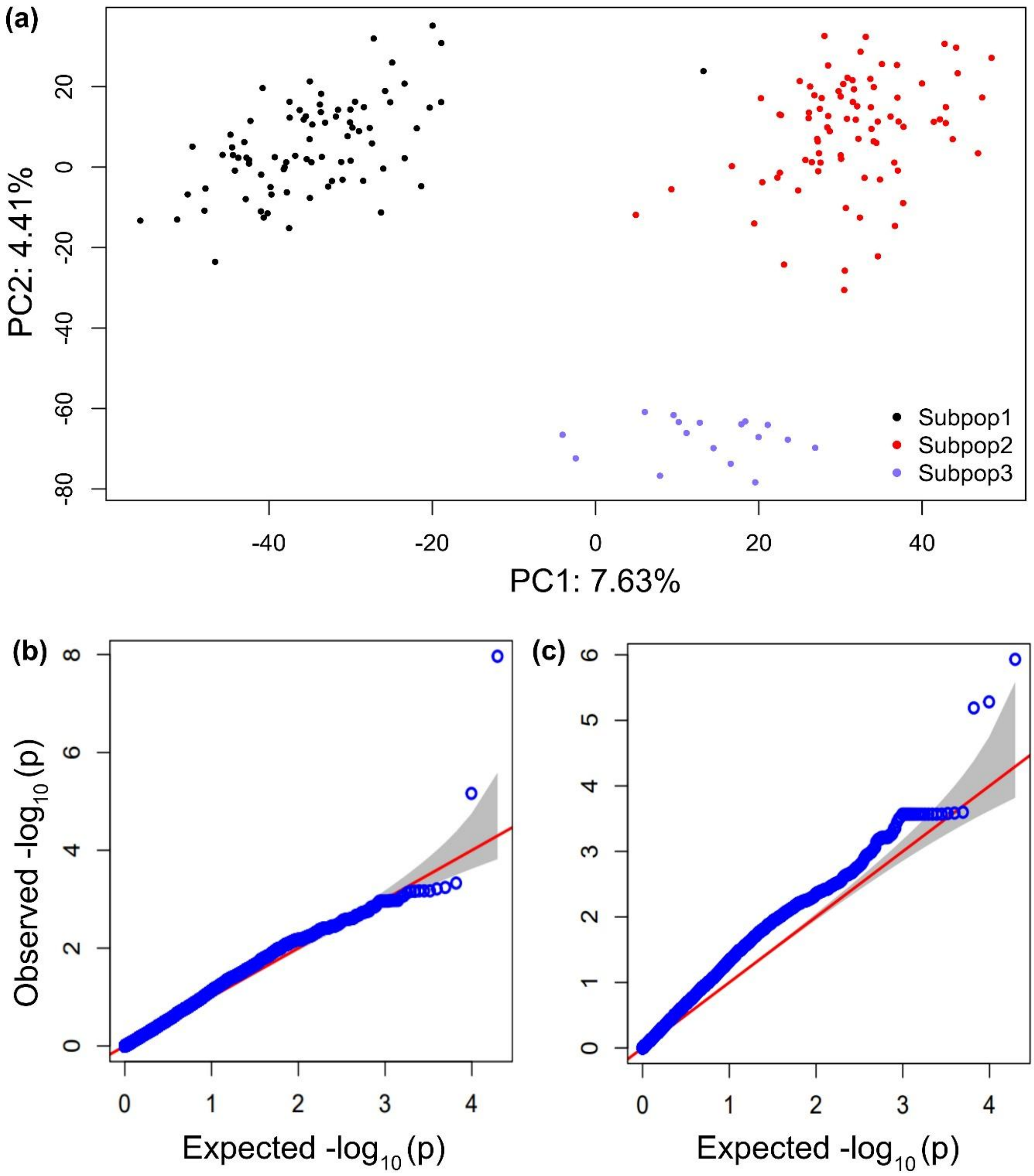

Fig. S1 (a) PCA plot with first two PCs showing the structure of the KE testcross population for FSR severity **(b)** Q-Q plot showing correction in population structure in FSR severity **(c)** Q-Q plot without correction in population structure for FSR severity

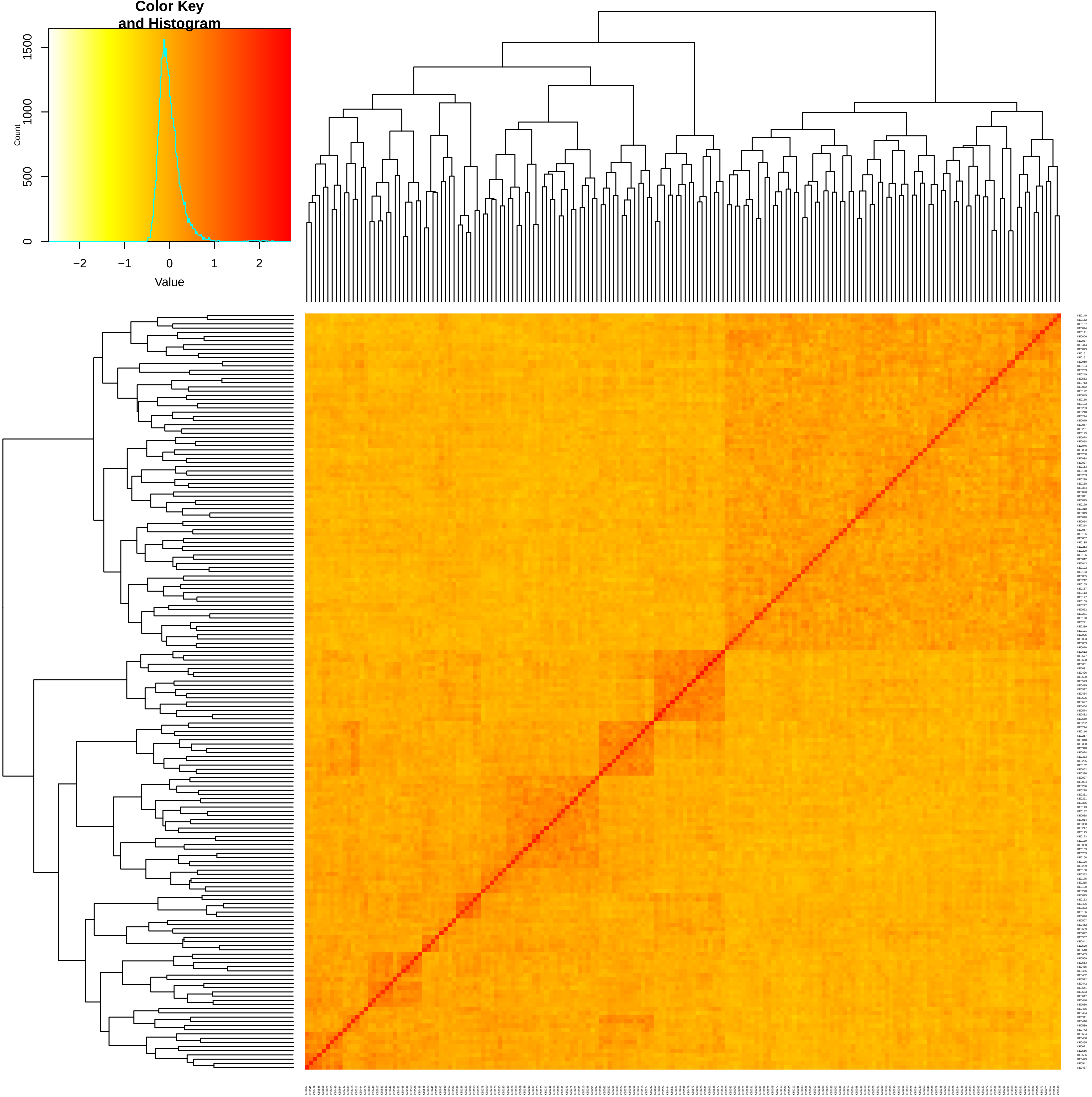

Fig. S2. Kinship heatmap of 180 KE DH population

Table S1 Selection of principal components based on BIC values

No. PCs/Covariates	BIC (larger is better) - Schwarz 1978	Log Likelihood Function Value
0	-736.045	-728.255
1	-737.64	-727.254
2	-738.178	-725.196
3	-739.684	-724.105
4	-740.254	-722.079
5	-741.123	-720.352
6	-741.771	-718.402
7	-735.379	-709.415
8	-737.216	-708.655
9	-735.455	-704.297
10	-737.095	-703.34

Table S2 Predicted candidate genes associated with FSR severity, DS and PH in the genomic regions detected by GWAS

Trait	Marker	Genomic region	Gene ID	Annotation	Gene ontology
**FSR severity**	ZmSYNBREED_10522_344	Chr1: 17,609,594-17,615,130	Zm00001d027918	Unknown	—
		Chr1: 17,827,591-17,829,479	Zm00001d027927	Unknown	—
		Chr1: 17,839,738-17,840,866	Zm00001d027929	Ethylene-responsive element binding protein 2	DNA-binding transcription factor activity, transcription cis-regulatory region binding.
		Chr1: 17,918,578-17,921,737	Zm00001d027930	Unknown	—
		Chr1: 17,944,810-17,962,531	Zm00001d027934	Leucine-rich transmembrane protein kinase 1	Protein serine/threonine kinase activity, protein binding, ATP binding, protein phosphorylation
		Chr1: 17,977,813-17,985,594	Zm00001d027937	Pentatricopeptide repeat protein 12	Chloroplast accumulation
		Chr1: 18,004,884-18,006,730	Zm00001d027939	B3 domain-containing protein	DNA binding
	ZmSYNBREED_51479_551	Chr6: 8,508,247-8,512,593	Zm00001d035182	Uncharacterized	—
		Chr6: 8,568,724-8,572,976	Zm00001d035186	Electron carrier/iron binding protein	—
		Chr6: 8,562,525-8,568,023	Zm00001d035185	Endoribonuclease E-like protein	—
**DS**	ZmSYNBREED_31239_115	Chr3: 17,379,140-17,381,376	Zm00001d039878	Uncharacterized	Response to stress, involved in response to abscisic acid
	ZmSYNBREED_66242_938	Chr8: 168,883,530-168,888,030	Zm00001d012146	U-box domain-containing protein 7	Protein binding, ubiquitin-protein transferase activity
		Chr8: 169,382,728-169,386,450	Zm00001d012167	Protein IQ-domain 14	Protein binding
		Chr8: 169,165,352-169,173,482	Zm00001d012159	Beta-glucosidase 4-like	Beta-glucosidase activity
	ZmSYNBREED_65065_837	Chr8: 126,282,952-126,285,155	Zm00001d010732	Rhogene7	GTPase activity, protein binding, GTP binding, glycolytic process, intracellular protein transport, nucleocytoplasmic transport, oxidation-reduction process, cellular carbohydrate metabolic process.

		Chr8:126,502,112-126,503,641	Zm00001d010743	calcineurin B-like-interacting protein kinase19	ATP binding, protein serine/threonine kinase activity
		Chr8:126,238,922-126,239,749	Zm00001d010730	TCP-transcription factor 7	Sequence specific DNA binding, DNA-binding transcription factor activity
		Chr8:126,505,809-126,509,764	Zm00001d010744	Uncharacterized	Gamma-glutamyl- peptidase activity
		Chr8:126,262,696-126,268,370	Zm00001d010731	import inner membrane translocase subunit TIM50	—
		Chr8:126,443,231-126,443,927	Zm00001d010741	Unknown	—
	ZmSYNBREED_65628_540	Chr8: 146,317,656-146,321,756	Zm00001d011308	Ubiquitin-like superfamily protein	Protein binding
		Chr8:146,174,056-146,176,755	Zm00001d011301	starch branching enzyme3	Leaf senescence, cation binding, starch biosynthesis process, growth
		Chr8:146,108,765-146,113,773	Zm00001d011298	putative C3HC zinc finger-like family protein	Zinc ion binding
		Chr8: 146,128,641-146,131,503	Zm00001d011299	sweet6b	Sugar transmembrane transport activity
		Chr8: 146,062,014-146,075,281	Zm00001d011294	Unknown	—
		Chr8: 146,093,648-146,095,671	Zm00001d011297	putative MYB DNA-binding domain superfamily protein	DNA binding and zinc ion binding: involved in response to ethylene and gibberellin
		Chr8:146,192,052-146,196,828	Zm00001d011304	Unknown	—
**PH**	ZmSYNBREED_65326_781	Chr8:136,302,778-136,306,209	Zm00001d010998	WUSCHEL-related homeobox 3b	DNA binding, DNA binding transcription factor activity
		Chr8:136,504,799-136,508,752	Zm00001d011005	arginine/serine-rich splicing factor RS2Z39 transcript VI	RNA binding, Zinc ion binding

		Chr8:136,509,382-136,511,838	Zm00001d011006	ABC transporter G family member 16	ABC-type transporter activity, ATP binding, ATP hydrolysis activity
		Chr8:136,569,078-136,571,570	Zm00001d011011	Unknown	—
		Chr8:136,138,654-136,139,747	Zm00001d010993	Unknown	—
	ZmSYNBREED_65128_528	Chr8:129,538,321-129,541,025	Zm00001d010838	zinc-regulated, iron-regulated transporter-like protein6	Zinc ion transmembrane transporter activity
		Chr8:129,331,385-129,334,443	Zm00001d010834	Alfin-like-transcription factor 9	Transcription cis-regulatory region binding, histone binding, transcription coregulatory activity.
		Chr8:129,351,635-129,358,512	Zm00001d010835	arginyl-tRNA synthetase	ATP binding, arginine- tRNA ligase activity
